# Challenges in the treatment of late-identified untreated congenital adrenal hyperplasia due to CYP11B1 deficiency: Lessons from a developing country

**DOI:** 10.3389/fendo.2022.1015973

**Published:** 2022-12-15

**Authors:** Agustini Utari, Sultana M. H. Faradz, Annastasia Ediati, Tuula Rinne, Mahayu Dewi Ariani, Achmad Zulfa Juniarto, Stenvert L. S. Drop, Antonius E. van Herwaarden, Hedi L. Claahsen-van der Grinten

**Affiliations:** ^1^ Center for Biomedical Research (CEBIOR), Faculty of Medicine, Diponegoro University, Semarang, Indonesia; ^2^ Division of Pediatric Endocrinology, Department of Pediatrics, Faculty of Medicine, Diponegoro University, Semarang, Indonesia; ^3^ Faculty of Psychology, Diponegoro University, Semarang, Indonesia; ^4^ Department of Human Genetics, Radboud University Medical Center, Nijmegen, Netherlands; ^5^ Division of Pediatric Endocrinology, Sophia Children’s Hospital and Erasmus Medical Center, Rotterdam, Netherlands; ^6^ Department of Laboratory Medicine, Radboud University Medical Center, Nijmegen, Netherlands; ^7^ Division of Pediatric Endocrinology, Department of Pediatrics, Amalia Children’s Hospital, Radboud University Medical Center, Nijmegen, Netherlands

**Keywords:** congenital adrenal hyperplasia, CYP11B1 deficiency, differences (disorders) of sex development, developing country, guideline

## Abstract

**Background:**

Congenital Adrenal Hyperplasia (CAH) due to CYP11B1 is a rare autosomal recessive adrenal disorder that causes a decrease in cortisol production and accumulation of adrenal androgens and steroid precursors with mineralocorticoid activity. Clinical manifestations include cortisol deficiency, ambiguous genitalia in females (differences of sex development (DSD)), and hypertension. Medical treatment recommendations are well defined, consisting of glucocorticoid treatment to substitute glucocorticoid deficiency and consequently normalize adrenal androgen and precursors levels. Current guidelines also emphasize the need for specialized multidisciplinary DSD teams and psychosocial support. In many developing countries, care for DSD patients, especially when caused by an adrenal disease, is challenging due to the lack of infrastructure, knowledge, and medication.

**Objective:**

The study aims to report the conflicting decision-making process of medical treatment and sex assignment in late-identified CAH patients in developing countries.

**Methods:**

We describe the clinical and biochemical findings and the psychological assessment of five affected but untreated family members with CAH due to CYP11B1 deficiency.

**Results:**

All patients had a 46,XX karyotype, ambiguous genitalia, low cortisol levels, and hypertension. Two identified as males, two as females, and one had undecided gender. The patients were counselled that refusing treatment will lead to infertility and the potential risk of developing Addisonian crisis and severe hypertension. However, all 46,XX CAH males refused treatment with glucocorticoids due to the expected lowering of adrenal androgens as their main source of testosterone. None of the patients developed Addisonian crisis, probably due to some residual cortisol activity and glucocorticoid activity of elevated adrenal steroid precursors.

**Conclusion:**

Medical treatment and sex assignment in late-identified 46,XX CAH patients in Indonesia may often depend on local and cultural factors. The management of DSD conditions may have to be individualized and integrated into the psychological and social context of the affected family.

## Introduction

Congenital adrenal hyperplasia (CAH) belongs to a group of inherited disorders of the adrenal cortex affecting cortisol biosynthesis due to the lack of one of the enzymes involved in adrenal steroid synthesis. CYP11B1 (11β-hydroxylase) deficiency, caused by a mutation in the *CYP11B1* gene, is a rare form of CAH, accounting for 5-8% of CAH cases with a global estimated prevalence of 1: 100.000 in live births (1), but in Indonesia, the prevalence has not yet been reported. Cortisol deficiency leads to a lack of negative feedback at the pituitary level and consequently increases adrenocorticotropic hormone (ACTH) production, resulting in the overproduction of steroid precursors before the enzymatic block. Consequently, adrenal androgen and mineralocorticoid synthesis is increased leading to ambiguous external genitalia in affected 46,XX neonates and chronic hypertension in both sexes (1–3). Therefore, CYP11B1 deficiency belongs to the umbrella term of differences (disorders) of sex development (DSD).

The diagnosis of CYP11B1 deficiency is based on clinical features, hormonal analysis and can be confirmed by mutation analysis. Biochemical analysis shows significantly increased levels of 11-deoxycortisol, deoxycorticosterone (DOC), and androstenedione(A) with normal or slightly increased 17-hydroxyprogesterone (17OHP) levels. Cortisol levels are generally low, and renin levels are usually suppressed or within the lower range (4, 5). Hypokalemia is an uncommon finding, particularly in infants (5). Delayed diagnosis and treatment in patients with CYP11B1 deficiency may lead to further virilization (clitoris hypertrophy) and psychological problems such as gender dysphoria in 46,XX individuals as well as precocious puberty in 46,XX and 46,XY individuals. Moreover, adolescents develop severe hypertension (1, 2, 6). Patients may develop Addisonian crises due to cortisol deficiency, but the prevalence of adrenal crises is not reported.

In general, CAH guidelines recommend treatment with hydrocortisone to substitute for cortisol deficiency, consequently lowering ACTH and androgen concentrations. In CYP11B1 deficiency, a decrease in ACTH also results in a decrease of mineralocorticoid precursors preventing the development of hypertension (1). Furthermore, patients require an increase of glucocorticoid dosage in situations of severe illness. Therefore, patients have to be educated carefully about their condition. Health care providers have to be aware of the clinical condition to treat patients adequately especially in emergency situations. Current guidelines recommend follow-up of DSD patients in specialized centres that provide psychosocial support for patients and their families, especially in sex assignment and the possibility of genital corrective surgery (7). It should be noted that these guidelines generally do not take inter-country or inter-cultural differences into account.

In Indonesia, as in many developing countries, care for DSD patients, particularly CAH patients, is poorly developed. From our local database in Central Java 12.8% of CAH children die before the age of 6 years (8), in contrast to western countries where nearly all CAH children reach adulthood without serious complications. In addition, patients are often undetected and in the most severe cases they die already in the neonatal period, as a neonatal screening program for CAH is not implemented and access to medication such as hydrocortisone is limited. Furthermore, biochemical equipment and medical specialists with special expertise in CAH are insufficiently available and patient compliance to treatment is poor due to poor social economic conditions and low (parental) education.

This study reports a large Indonesian family with late-identified, untreated CYP11B1 deficiency. We describe a marked variation in genital phenotype, including gender preference, strongly influenced by local cultural factors and a history of limited access to medical care. We discuss the conflicting decision-making process that depends strongly on local and cultural factors, and show that treatment with glucocorticoids, as recommended by current international guidelines, is challenging to be implemented in these adrenal DSD conditions.

## Materials and methods

We identified an Indonesian family with four children and one relative with 46,XX DSD caused by untreated CAH due to CYP11B1 deficiency. All patients were examined by a pediatric endocrinologist (AU). Cortisol and adrenal steroid precursors were measured before and 60 minutes after a standard ACTH stimulation test using tetracosactide acetate (synacthen**^®^**) 250 ug iv injection following the published standard protocol (9). The diagnosis was confirmed by molecular analysis. This study was approved by the ethical committee of Faculty of Medicine, Diponegoro University, Indonesia (No.713/EC/FK-RSDK/2016). Written and oral informed consent was obtained from the patients and their parents. Gender-specific annotations as “she/he” or “male/female” are based on the current gender identity.

### Biochemical analysis

All blood samples were taken in the morning before 9 a.m. after overnight fasting. All patients were untreated and did not take any additional medication. Cortisol and adrenal steroid precursors were measured before and 60 minutes after the standard ACTH stimulation test using synacthen 250 ug iv injection following the published standard protocol (9). Blood samples were immediately centrifuged, and serum were stored at -80°C until shipment occurred. The specimen was transported on dry ice to the Radboud University Nijmegen Medical Centre (Radboudumc), The Netherlands, within approximately 24-28 hours. Serum Cortisol, S, 17-OHP, DOC, A, and testosterone concentrations were measured in serum using Liquid Chromatography- Tandem Mass Spectometry (LC-MS/MS) as described in Ter Horst et al., 2016 (10).

Serum renin levels were measured in EDTA plasma using an immunoradiometric assay from CISbio (genIII). Serum potassium and sodium levels were measured in heparin plasma by ion-selective electrodes on a c8000 random access analyzer from Roche.

### Molecular analysis

To confirm the diagnosis, we performed molecular analysis of the *CYP11B1* gene of these patients in the Genome diagnostic laboratory of Radboudumc, The Netherlands. Genomic DNA (gDNA) was extracted from peripheral blood leukocytes of the patients according to standard procedures (in Indonesia). Nine coding exons and flanking parts of the intronic sequences of the *CYP11B1* gene were amplified by polymerase chain reaction (PCR). The amplified PCR products were purified and sequenced on ABI 3730XL DNA sequencer using the BigDye Terminator v1.1 Cycle Sequencing Kit. The sequencing results were compared with the reference sequence GenBank NM_000497 (CYP11B1 cdna).

### Psychological evaluation

The psychological evaluation was conducted by a clinical psychologist (AE) in the Center for Biomedical Research (CEBIOR), Faculty of Medicine Diponegoro University (FMDU), Semarang, Indonesia. Individual interviews were conducted with all patients and parents during their initial visit and follow-up meetings to assess gender identity and development, discuss their concerns and difficulties in coping with the CAH condition in their daily lives, particularly for adolescent patients or children entering school periods. Parents were interviewed to assess their wishes and concern related to their child’s gender identity and gender assignment/reassignment. The possibility of social stigmatization or social influence affecting parents’ decision-making on gender assignment were also explored.

## Results

### General background of the family

**Figure 1:**
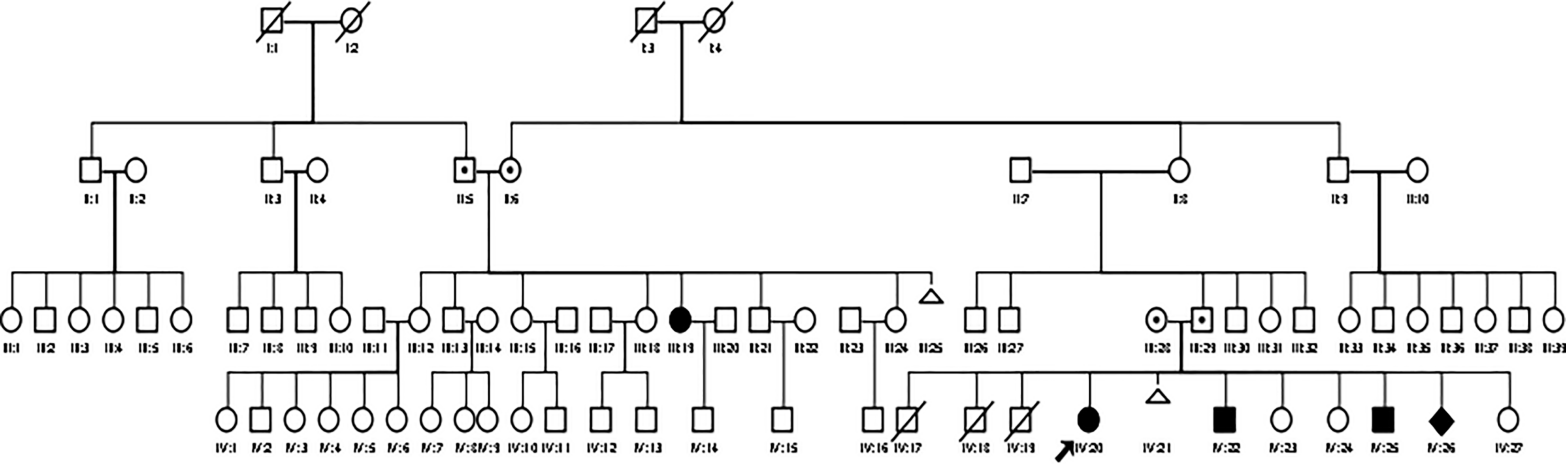
A large pedigree of the Indonesian family with CYP11B1 deficiency. Circles and squares indicate female and male gender identity, respectively, while diamond indicate sex undetermined.

We present a family with four affected children and one affected relative (cousin of the father) in a large pedigree (see **Figure 1**). There is no documented consanguinity of the parents, but they originally came from the same isolated region. The family has a low educational and social-economic background and lives in a small village with limited access to medical healthcare and public transportation. The parents did not finish elementary school. The father works as a farmer and is the decision maker in this family. The parents apply the religious practice in coping with the children’s conditions. The family had 11 children; three children (males) were born prematurely without affected genitalia and died in the neonatal period, most likely due to prematurity. There was one miscarriage. The family was referred to the DSD team of the Diponegoro University in Semarang because of an observed male physical appearance of the oldest female sibling aged 20 years (Patient 1, IV:20, see **Figure 1**). Local government officials in the village initiated seeking medical consultation due to doubts about her gender, and also took the remaining family members to our center for examination.

**Figure 2:**
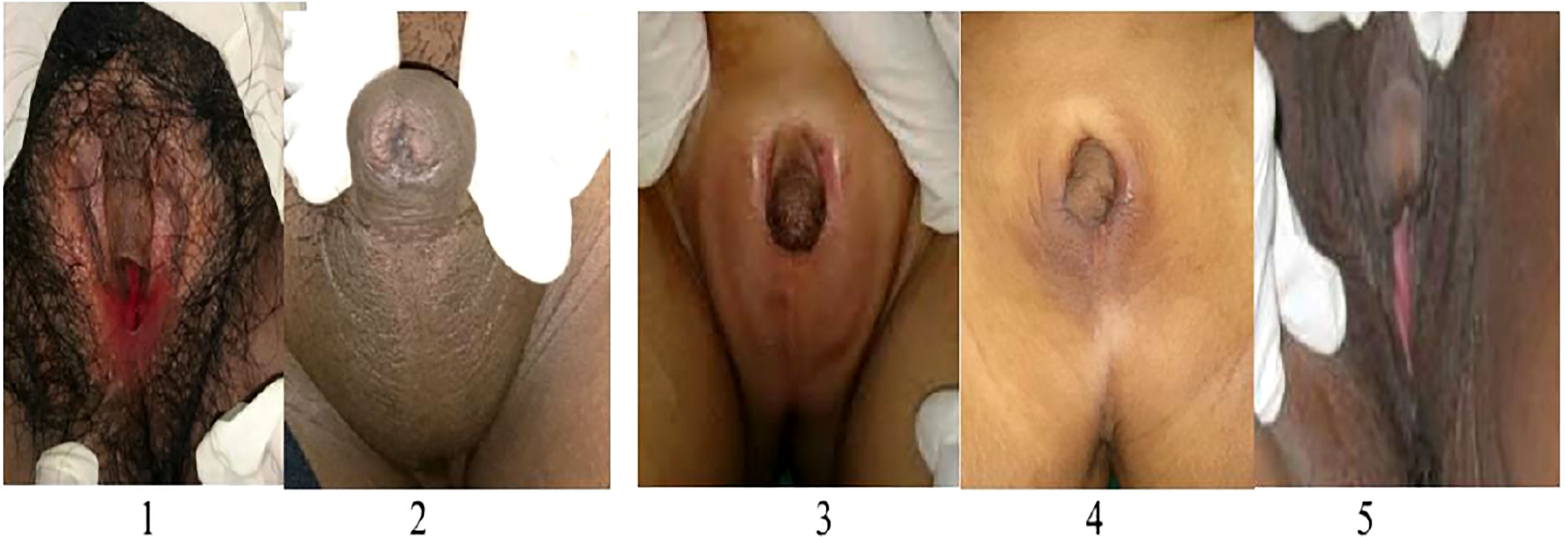
Phenotype of external genitalia in a family with CYP11B1 deficiency. Patient 1 and 5 identified as females and presented with clitoromegaly and posterior labial fusion (Prader 3), Patient 2,3, and 4 identified as males had complete labial fusion and a urethral meatus at the tip of phallus (Prader 5).

Here we give a detailed description of the phenotype, the clinical symptoms, and treatment of all five affected patients (see **Table 1**), including the variation of genitalia in the affected patients (see **Figure 2**). All patients had a 46,XX karyotype.

**Table 1:** Clinical characteristics and initial biochemical analysis in a family with CAH due to CYP11B1 deficiency.

Variables	Patient 1 (IV;20)	Patient 2 (IV:22)	Patient 3 (IV:25)	Patient 4 (IV:26)	Patient 5 (III:19)
**CLINICAL FEATURES**					
Age (y)	20	13	6	4	28
Gender identity	Female	Male	Male	Undecided	Female
Karyotype	46,XX	46,XX	46,XX	46,XX	46,XX
Weight (kg)	69.2	30	18.9	12.2	49.7
Height (cm)	145	135	108.4	90.3	147.9
HAZ score*	-2.79	-3.18	-1.19	-2.36	-2.3
Genetic potential for height**	137.6-154.6	137.6-154.6	137.6-154.6	137.6-154.6	N/A
Bone Age acceleration	N/A	+3y	+3y	+6m	N/A
BMI (kg/m2)***	32.7	16.5	16.1	15	22.7
BP (mmHg)****	136/100	132/80 (> p95)	100/70 (p90-p95)	90/60 (<p 90)	150/105
Tanner Stage	M2P5	M2P5	M1P1	M1P1	M4P5
Hyperpigmentation	Yes	Yes	No	No	Yes
Acne	Yes	Yes	No	No	Yes
Menarche (y)	15	N/A	N/A	N/A	18
Menstrual cycles	Irregular	N/A	N/A	N/A	Irregular
Low/deep voice	Yes	Yes	Yes	No	Yes
Hirsutism (mFG)******	23	3	0	0	17
Prader stage	3	5	5	5	3 #
Phallus length (cm)	3.7	4.7	2.8	2.3	5.7 #
Anogenital distance(cm)	7	10.4	8.5	6.7	N/A
**HORMONAL ANALYSIS**					
Cortisol (nmol/l) (1)	218	179	150	202	164
Cortisol (nmol/l) (2)	255	180	155	202	163
Reference values	(190-550)				
S (nmol/l) (1)	381	196	301	417	275
S (nmol/l) (2)	430	657	457	567	297
Reference values	(0.2-4.3)				
17 OHP (nmol/l) (1)	17	6	11	13	10.6
17 OHP (nmol/l) (2)	19.7	20.4	19.4	22.4	12.5
Reference values	(0.45-3.28)	(0.45-3.8)	(0.2-7.4)	(0.2-7.4)	(0.45-3.28)
A (nmol/l) (1)	68.6	35.9	19.6	18	50.4
A (nmol/l) (2)	74.2	67.6	22.6	19.3	51.3
	(R:1.22-7.16)	(R:0.52-4.78)	(R:0.07-0.98)	(R:0.07-0.98)	(R: 1.22-7.16)
Testosterone(nmol/l) (1)	6.23	6.63	2.89	1.96	10.8
Testosterone(nmol/l) (2)	6.52	7.82	3.83	2.21	11.1
	(R:0.52-2.0)	(R:0.52-2.0)	(R:0.02-0.3)	(R:0.2-0.3)	(R:0.52-2.0)
Renin (mU/l)	< 3	7.9	<3	5.9	9.7
	(R:4.4-85)	(R:4.4-85)	(R:4.4-85)	(R:4.4-85)	(R:4.4-85)
Na (nmol/)	151	151	153	148	150
	(R:135-145)	(R:135-145)	(R:135-145)	(R:135-145)	(R:135-145)
K (nmol/l)	3.85	N/A	3.66	3.55	4.17
	(R:3.5-5)	(R:3.5-5)	(R:3.5-5)	(R:3.5-5)	(R:3.5-5)

BMI, Body Mass Index; y, years; BP, blood pressure; R, reference value; mFG, modified Ferriman-Gallwey; S, 11-deoxycortisol; 17 OHP, 17-hydroxyprogresterone; A, Androstenedione; Na, sodium; K, potassium.

All blood samples for hormonal analysis were taken before 9:00 am.

(1) baseline measurement; (2) post ACTH stimulation.

# the data from medical record before she underwent a clitoroplasty and vaginoplasty.

*HAZ is height-for-age Z score based on WHO anthro 2007 for girls.

**Genetic potential for height is calculated using formula: (Father’s height - 13 + Mother’s height)/2 ± 8.5 cm.

***BMI for children based on World Health Organization (WHO) growth chart for girls.

****Clinical practice guideline for screening and management of high blood pressure in children and adolescents(11).

****Ferriman-Gallwey Score for hirsutism (range 0-36), >8 considered as hirsutism.

### Description of individual patients

#### Patient 1 (IV:20)

This 20-year-old woman was referred to our clinic due to an observed male physical appearance. She had a deepening voice and a mustache, beard, and acne had progressively developed starting at the age of 12 years. She had her menarche at the age of 15 years, but her menstruation had subsequently been irregular. No symptoms suggested salt-wasting crises, cortisol deficiency, or Addisonian crises.

Her psychological profile recorded that she was raised as female, developed a female gender identity, and female gender role behavior. However, people often misidentified her as a male due to her low/deep voice, beard, and conspicuous muscularity. Over time, she began to doubt her female gender identity and limited her social contacts. She was sexually attracted to males only but never had a sexual relationship. She often masturbated but never had an orgasm.

At physical examination at 20-years old, the patient was obese (BMI 32.7 kg/m2), with severe hirsutism (Ferriman-Gallwey Score of 23/36), hyperpigmentation of skin and genitalia, and hypertension (ambulatory blood pressure of 136/100 mmHg) that was not detected before. Furthermore, she had an enlarged clitoris and posterior labial fusion (Prader 3) (**Figure 2.1**).

The clinical diagnosis of CYP11B1 deficiency was made, and glucocorticoid treatment was started with prednisone 2.5 mg twice daily to reduce adrenal androgens and normalize blood pressure. In addition, she was educated about the increase of glucocorticoid doses in case of emergency.

We conducted regular three-months reviews. After two years, she was moderately compliant in following the prescribed treatment. Blood pressure normalized, however, the BMI increased. She attained softer and lighter skin and regular menstruation which made her more confident with her more feminine appearance. However, she requested to undergo feminizing surgery only when she has plans to marry.

#### Patient 2 (IV:22)

Genital ambiguity was identified at birth by the traditional midwife, but the father decided not to seek medical help. Instead, the midwife told the parents that the child was a *“kuming”* (local terms for an ambiguous or transsexual person). At the age of 7 years, the father assigned a male gender based on his observations because a birth certificate was required as part of school registration. There were no complaints suggesting cortisol deficiency. Hypertension (120/90 mmHg) (**Table 1**) was first detected at the age of 11 years.

At the initial visit to our center when the child was 13 years old, he identified himself as a male. Psychological evaluation at the initial visit revealed that the child had developed a male gender role behavior and showed male-type preferences in clothing and play activities. However, he preferred to clean his home, which is considered a feminine task. He refused contact with peers and seemed to have difficulty expressing his feelings. He was bullied at school because of his unclear doubts about his gender, and he had difficulties in academic adjustment.

At the age of 15 years, he reported no gender confusion or dissatisfaction. He appeared to be happy living as a male. He moved to another school at the father’s insistence due to his poor academic achievement. No bullying was reported at the new school. Although his parents were not concerned about their child’s gender identity, both parents and the child were aware of infertility with the male gender. The boy reported painful breast development. Physical examination at the initial visit revealed short stature, severe acne, breast budding (Tanner 2), hyperpigmentation, hypertension, and genitalia at Prader stage 5. After two years of follow-up, the breast developed into Tanner 3. At the age of 13 years, glucocorticoid treatment was suggested by a pediatric endocrinologist to treat the hypertension but as it was explained that the expected decrease in adrenal androgens would result in estrogen production by the ovaries with consequent feminization, breast development, and menstruation, he refused treatment. At age 15, he started with anti-hypertensive medication using a calcium channel blocker (amlodipine) but with poor compliance. He is followed up in a primary health care center nearby without experience in DSD conditions.

#### Patient 3 (IV:25)

This 6-years-old child was raised as a male. He had no complaints and no history of Addisonian crises or hypertension. Like his older brother (patient 2), genital ambiguity was identified at birth by the traditional midwife. Sex assignment as a male was made by the father directly after birth. Psychological evaluation at age 6 showed male-type gender role behavior and male-type preferences in clothing, playmate, toys, and play activities. Unlike his older brother, this child had many social contacts and seemed to accept his appearance. After two years of follow-up, the child still looked happy with his male gender. At physical examination at the age of 6 years, the degree of virilization of the genitalia was Prader stage 5. His blood pressure was 100/70 mmHg (p90-p95) (11). After two years of follow-up, his blood pressure further increased to 125/90 mmHg. The growth velocity was increased (14.7 cm in two years). A pediatric endocrinologist discussed with the father about the glucocorticoid treatment that can help improve the hypertension. He declined glucocorticoid medication because a decrease in androgens would cause feminization of the child. The prescription of anti-hypertensive medication was considered but was refused by the parent.

#### Patient 4 (IV:26)

This 4-years-old-child was born with ambiguous genitalia but was otherwise healthy without any complaints. The father had not assigned sex at the patient’s age of 4 because he still had doubts about the child’s gender. According to the father, the child had soft skin and female-type behavior, which seemed to fit more to a female gender than a male gender, but he decided to take more time for observation. Father gave a unisex name to the child. During our observation, the child consistently looked happy with sufficient social contact. After two years follow-up, the child at age 6 had identified himself as male gender and exhibited male-type gender behavior. Although the father had not assigned gender, the parenting practices showed that the parents treated the child as a boy. He fully understood that the child had female chromosomes and female reproductive structures, but he observed that the child already had male secondary sex characteristics (voice, hair, muscular body) as a sign of masculinity. Physical examination at the age of 4 showed no hyperpigmentation and no hypertension (90/60mmHg). The child had a phallus of 2.3 cm with complete labioscrotal fusion (Prader 5). Also, in this child, parents were counselled about infertility in a male gender role. A pediatric endocrinologist discussed the glucocorticoid treatment for this child, but the father again decided not to start treatment because of the same reasons described in the other children.

#### Patient 5 (III:19)

This patient was a cousin of the father. She was born with ambiguous genitalia, and the traditional midwife advised the parents to assign female sex. She had spontaneous pubertal development but delayed menarche at the age of 18 years. Peers frequently bullied her because of her low voice and masculine features. She was sexually attracted to men only. Her medical report (pre-surgery) described clitoromegaly and posterior labial fusion (Prader 3). Previously she was diagnosed with CAH due to 21-hydroxylase deficiency based on the increased 17-OHP concentrations. At the age of 22, she decided to undergo clitoroplasty and vaginoplasty but was lost to follow-up after surgery. A psychological evaluation revealed that she had always identified as female and had never experienced gender dysphoria. She reported unpleasant experiences of being mistakenly identified as a male due to her deep voice and muscular body.

At the age of 27 years, she married a widower with three adolescent children who lived 300 km from home. She reported being satisfied with her sexual functioning despite the infrequency of her meetings with her husband. She experienced a lower social position due to having CAH and desired to have a child, which would as well increase her social status. Physical examination at the age of 28 years revealed hypertension (150/105 mmHg), hyperpigmentation, hirsutism, and acne. She was treated with prednisone 2.5 mg twice a day in an attempt to reduce adrenal androgen levels. After starting treatment, she felt that her body weakened. Therefore, compliance was poor. After repeated education, her compliance improved, her blood pressure normalized, and she became pregnant within six months. She delivered a male infant by Caesarean section with normal male appearance. After becoming a mother, she felt that her standing in the family and neighborhood markedly improved.

### Biochemical analysis

In all patients basal and ACTH stimulated 11-deoxycortisol and androstenedione levels were highly increased, basal 17-OHP levels were slightly increased, and renin concentrations were suppressed or within the lower normal range. Serum sodium levels were elevated, and serum potassium levels were within the normal range. Cortisol concentrations before and after ACTH stimulation remained below 500 nmol/l indicating biochemical cortisol deficiency.

### Molecular genetics

The cytogenetic analysis showed a 46,XX karyotype in all patients. A homozygous pathogenic mutation was detected in the *CYP11B1* gene (NM_000497.3) by DNA sequence analysis confirming the CYP11 deficiency. The detected mutation c.799G>A affects the last nucleotide of exon 4 and disrupts the consensus splice donor site (12). No other pathogenic mutations or variants of uncertain significance were detected.

## Discussion

Here we report the medical histories and clinical decision management in a family with five affected but untreated members only recently diagnosed with DSD due to CYP11B1 deficiency. To our knowledge, this is the first description of 5 members of a large family with untreated 11-OHD in a developing country. All affected individuals had a 46,XX karyotype and were born with various degrees of ambiguous genitalia. Three 46,XX individuals raised as males and two as females. Besides the decision on sex assignment in this DSD condition, the decision about hormonal treatment with glucocorticoid that will influence androgen status is challenging. In general, treatment of CYP11B1 deficiency consists of glucocorticoids substitution to treat cortisol deficiency. By restoring the negative feedback to ACTH secretion, adrenal androgen production, as well as the production of aldosterone precursors, will be reduced thereby normalizing mineralocorticoid-induced blood pressure (3, 5). Although international guidelines give clear recommendations about this treatment, the decision-making process in Indonesia as a model for developing countries is complicated and strongly influenced not only by limited access to medication and medical care but also by local socioeconomic and cultural factors (13).

In the last years, changing ethical and legal norms in managing differences of sex development have also been observed. Sudai describes a change in attitude from the gender natural identity theory allowing early decision-making in infants with DSD to a more restricted approach where patient’s autonomy and the decision became more important (14). Current guidelines emphasize the need for highly specialized multidisciplinary teams and psychosocial support for patients and their families. In western countries, this approach is successfully implemented in expert centers taking care of patients with DSD, which are not well established in developing countries.

Three main ethical principles prevail in sex assignment in DSD patients due to CAH and counseling to determine the use of glucocorticoids and/or to perform uro-surgical interventions (15).

### The well-being of the child/adult, including body integrity and quality of life

Besides the challenging decisions about the need to perform genital surgery, 46,XX CAH individuals due to CYP11B1 deficiency, if left untreated, develop hypertension and progressive virilization of the external genitalia and a male appearance with serious physical and psychosexual consequences, such as disturbed pubertal development, menstrual disturbances, decreased fecundity. The treatment of choice generally consists of glucocorticoids such as prednisone which is not expensive and nowadays available even in remote areas. In one of our patients who identified herself as female and agreed to treatment, prednisone administration led to an improvement of the quality of life and gender dysphoria due to diminishing virilization and restoration of the menstrual cycle and even to pregnancy. In addition, blood pressure normalized, thereby decreasing the risk of long-term complications (3, 5). Clitoral reduction and vaginoplasty is a complex and invasive procedure requiring expertise and adequate medical treatment of the patient, and careful long-term follow-up (16). In contrast, in 46,XX CAH patients who identify as males, treatment with glucocorticoid substitution with lowering of adrenal androgen concentration will lead to signs of hypogonadism, such as reduction in muscle strength as described in one of the patients. Furthermore, by lowering adrenal androgens levels, the pituitary-gonadal axis, suppressed by the elevated adrenal androgens, will be activated. This will result in activation of the hypothalamic-pituitary-ovarian axis with breast development, menstruation, and risk of developing gender dysphoria. Therefore, as in our patients, glucocorticoid treatment is mostly refused by 46,XX patients who identify as males. The primary goal in these patients is not to suppress androgens but to prevent Addisonian crises and treat chronic hypertension. Anti-hypertensive medication such as aldosterone antagonists, potassium-sparing diuretics, or calcium channel blockers can control blood pressure without interfering with androgen concentrations (3, 5).

According to the current standards, chronic glucocorticoid treatment and a stress scheme in emergency situations are also recommended to prevent Addisonian crises in patients with biochemically confirmed cortisol deficiency. Interestingly, none of our untreated patients reported signs of Addisonian crises in the past. A recent *in-vitro* study showed that adrenal steroid precursors, which are strongly elevated in untreated CAH patients, have glucocorticoids activity that may partly compensate for cortisol deficiency (17). Treatment with glucocorticoid may probably even increase the risk of Addisonian crises by lowering steroid precursors concentrations. Lack of medical infrastructure and specific education of patients and health care providers about the use of a stress dose and also financial limitations may increase the mortality and morbidity in treated patients. Therefore, in developing countries, these factors have to be taken into account before starting glucocorticoid treatment.

### The right of the children/adult to participate in self-determining decisions

In general, in neonates with 46,XX CAH due to CYP11B1 deficiency, early glucocorticoid treatment is indicated without delay to prevent Addison crisis and hypertension. With this treatment also androgen levels will decrease. Genital surgery is mostly performed within the first year of life as there are no doubts about the female gender after lowering testosterone levels. However, there is an ongoing discussion about the early genital correction as it is postulated that children should participate in these decisions.

In our patients, the diagnosis was made late, and neither the parents nor the adolescent and adult patients actively sought medical help. The father delayed medical treatment and sex assignment until older age to be sure about the gender of the child based on behavior and physical appearance. The conflicting dilemma in choosing a male gender in 46,XX CAH patients is that starting with glucocorticoids will lower adrenal androgens, the main source of testosterone in these patients. In contrast, untreated 46,XX CAH male individuals will be infertile and have a potential risk to develop Addisonian crisis and severe hypertension. Our cases illustrate this conflicting dilemma of delayed diagnosis with already severe hypertension. The two patients who raised as females decided medical and uro-surgical treatment in adulthood.

### The family and parents’ relationship

The social, cultural and religious aspects of a family have to be respected. In the three younger patients, a delayed gender assignment had been indicated. Several studies report that 46,XX male CAH patients are satisfied with their male gender identity (18, 19). Abbas et al. reported on 3 adolescent 46,XX siblings with CYP11B1 CAH who were born with severe genital virilization and raised as males, One sib decided to change gender to female. After extensive psychological counselling her 2 sibs wished to continue to live as male (20). In contrast, previous studies in Indonesia showed that, particularly in untreated 46,XX CAH patients, male to female gender change occurred during adolescence and early adulthood (21). Thus, it will be important to provide long-term follow-up and psychological counseling. In many developing countries, medical care is often refused by DSD patients due to fear of stigmatization, as genital ambiguity is generally not accepted. Decisions are highly influenced by cultural and religious factors, and individual gender identity is often not considered. An essential factor in the decision-making process is that males have a higher social status, and infertility in males is more readily accepted (13). Recent studies in Indonesia showed that untreated patients with various forms of DSD, such as CAH, are at increased risk of stigmatization and experience more emotional and behavioral problems, especially social withdrawal, anxiety, and depression (22, 23). Low education, financial limitations, and cultural taboo in discussing sexuality (24) in the family complicate communication about the disease and its clinical management. Therefore, it is important to provide long-term individualized psychological and social skill training. In addition a structured education program offering integrated medical and psychological education using distant e-health involving also primary health care centers and local leaders, will help in dealing with the medical and psychosocial consequences of living with CAH (25).

**Figure 3:**
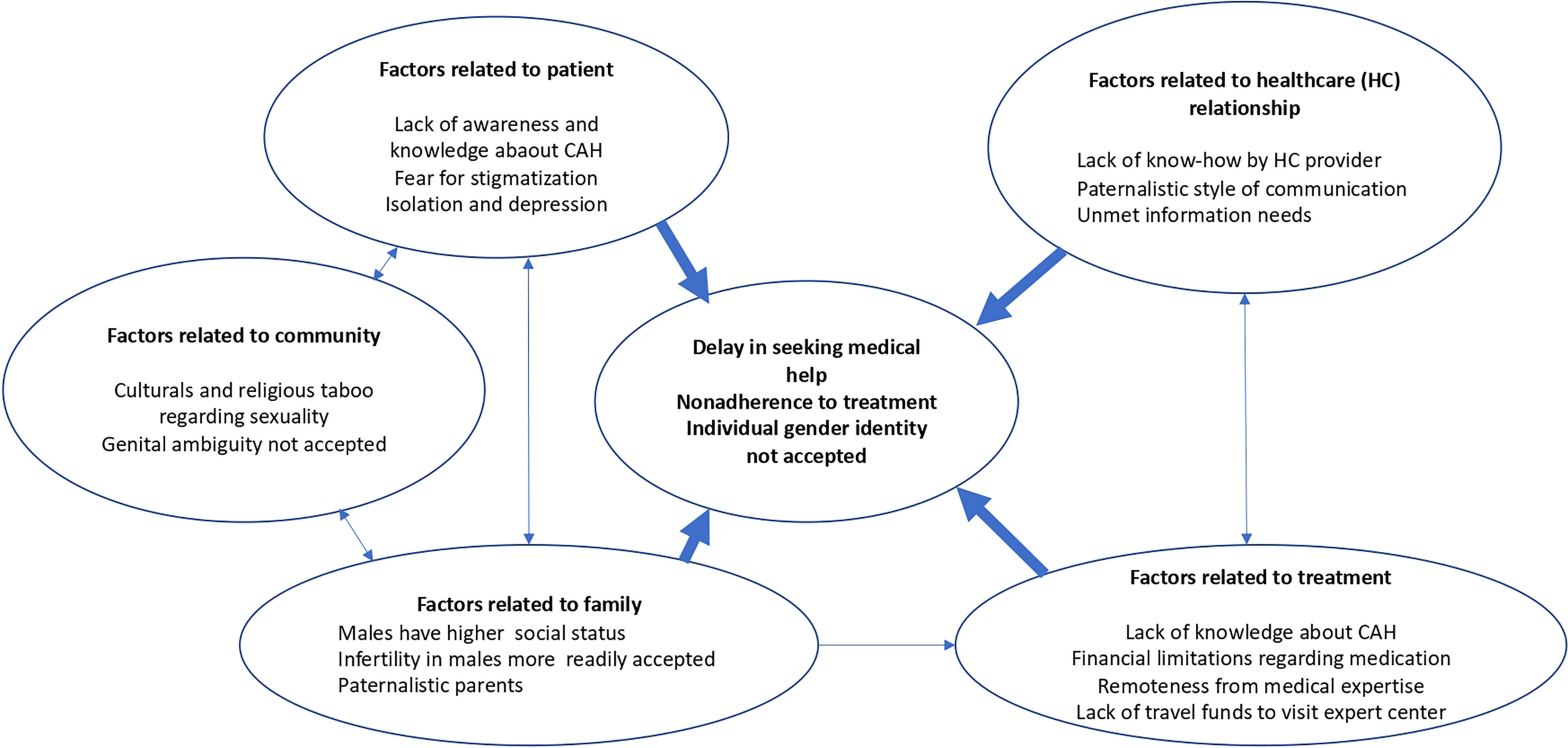
A practical model of factors related to delay in seeking medical help and nonadherence to treatment of patients with CAH in a developing country such as Indonesia.

We provide a model model that describes the main factors that affect use of care. Such a model may also be useful for other developing countries. (see **Figure 3**). We conclude that the management of DSD conditions should be individualized and integrated into the psychological and social context of the affected family. DSD due to adrenal diseases, such as CYP11B1 deficiency, leads to an additional conflicting discussion about the need for vital hormonal treatment. Adequate counseling of the patients, not only in terms of gender identity but also regarding long-term consequences, is important in the care of these patients.

## Data availability statement

The original contributions presented in the study are included in the article/supplementary material. Further inquiries can be directed to the corresponding author.

## Ethics statement

The studies involving human participants were reviewed and approved by The ethical committee of Faculty of Medicine, Diponegoro University, Indonesia (No.713/EC/FK-RSDK/2016). Written informed consent to participate in this study was provided by the participants’ legal guardian/next of kin. Written informed consent was obtained from the individual(s), and minor(s)’ legal guardian/next of kin, for the publication of any potentially identifiable images or data included in this article.

## Author contributions

AU, SF, SD, AE, AH and HC-G were involved in the conception and designed the study. AU, SF, MA, AJ collected the clinical data, AE collected the psychological data, TR analysed the mutation result, AH and HC-G analysed the hormonal results. All authors contributed to the article and approved the submitted version.
