# Corrigendum: Challenges in the treatment of late-identified untreated congenital adrenal hyperplasia due to CYP11B1 deficiency: Lessons from a developing country

**DOI:** 10.3389/fendo.2023.1210892

**Published:** 2023-05-04

**Authors:** Agustini Utari, Sultana M. H. Faradz, Annastasia Ediati, Tuula Rinne, Mahayu Dewi Ariani, Achmad Zulfa Juniarto, Stenvert L. S. Drop, Antonius E. van Herwaarden, Hedi L. Claahsen-van der Grinten

**Affiliations:** ^1^Center for Biomedical Research (CEBIOR), Faculty of Medicine, Diponegoro University, Semarang, Indonesia; ^2^Division of Pediatric Endocrinology, Department of Pediatrics, Faculty of Medicine, Diponegoro University, Semarang, Indonesia; ^3^Faculty of Psychology, Diponegoro University, Semarang, Indonesia; ^4^Department of Human Genetics, Radboud University Medical Center, Nijmegen, Netherlands; ^5^Division of Pediatric Endocrinology, Sophia Children’s Hospital and Erasmus Medical Center, Rotterdam, Netherlands; ^6^Department of Laboratory Medicine, Radboud University Medical Center, Nijmegen, Netherlands; ^7^Division of Pediatric Endocrinology, Department of Pediatrics, Amalia Children’s Hospital, Radboud University Medical Center, Nijmegen, Netherlands

**Keywords:** congenital adrenal hyperplasia, CYP11B1 deficiency, differences (disorders) of sex development, developing country, guideline

In the published article, there was an error in the Acknowledgments statement. The following sentence was missing from the statement and has now been added.

“The presented work resulted from collaboration made possible through the ESPE sponsored programme “ESPE Visiting Professorship” supported by Pfizer.”

The authors apologize for this error and state that this does not change the scientific conclusions of the article in any way. The original article has been updated.

